# Fragmentation behavior of minerals in vanadium slag through electric field intensified ultrasound cavitation^☆^

**DOI:** 10.1016/j.ultsonch.2026.108090

**Published:** 2026-09-30

**Authors:** Luyang Duan, Qianqian Ren, Shuang Cai, Xuelin Ma, Junguo Li, Yanan Zeng, Yajun Wang, Bao Liu

**Affiliations:** a College of Metallurgy and Energy, North China University of Science and Technology, Tangshan 063210, PR China; b Yanzhao Iron and Steel Laboratory, North China University of Science and Technology, Tangshan 063210, PR China

**Keywords:** Vanadium slag, Ultrasound cavitation, Electric field intensified ultrasound, Ultrasonic impact force, Mineral fragmentation

## Abstract

Ultrasound-assisted leaching is widely applied for extracting valuable metals from mineral resources. The cavitation effects generated by ultrasound treatment (UT) of a solution can fragment minerals that improve the leaching kinetics of the target metals. In this work, the fragmentation behavior of vanadium slag (VS) minerals through electric field intensified ultrasound treatment (EUT) was investigated. It was found that the ultrasonic impact force increased with decreasing ultrasound working distance (WD). Under the action of UT and EUT, the evolution of ultrasonic impact force as a function of ultrasound WD conformed to the natural exponential functional relationship. Inducing electric field into ultrasound field improved ultrasonic impact force, however, the ultrasonic impact force of EUT decayed more rapidly with increasing ultrasound WD. The silicate-wrapped structure of spinel in VS can be destroyed through EUT when the ultrasound WD was smaller than 1 cm. The broken extent of silicates was greater than that of spinel. The EUT had little effect on the fragmentation of the VS particles with particle size (PS) larger than 74 μm. While, it had significant effect on the reduction in PS and the enhancement of specific surface area (SSA) of the VS particles with PS smaller than 74 μm. The PS of the VS with PS smaller than 74 μm decreased by 61.2%, while that of the SSA increased by 18.3 times through EUT, which is beneficial to shortening the leaching time and improving the leaching efficiency of vanadium from the VS.

## 1 Introduction

Vanadium and its compounds are strategic resources widely used in production of high-quality steel [1], [2], aerospace materials [3], [4], vanadium redox flow batteries [5], [6], and catalysts [7], [8], [9], etc. It is reported that the global consumption of vanadium has exceeded 110 thousand tons, annually [10]. About 90% of vanadium was applied in manufacturing of high-quality steel in the past years. The vanadium redox flow batteries with the properties of large capacity, high safety and long service lifetime are recognized as a promising energy storage device. Statistically, the global consumption of vanadium in energy storage field had reached 8082 t in 2023, with a year-on-year growth of 59.95%. It is predicted that the global consumption of vanadium for vanadium redox flow batteries production will be 86000–105000  t V_2_O_5_, in 2040 [11]. Driving by the policy for all-vanadium redox flow batteries application, the demand for vanadium resources is urging.

Vanadium mainly exists in vanadium-titanium magnetite [12]. After smelting the vanadium-titanium magnetite in a blast furnace, and then blown into a converter, the vanadium slag (VS) with V_2_O_3_ contents of 7–12 wt% is obtained [13]. According to the statistic, about 76.2% of the vanadium products are manufactured using VS as the raw material in the world [14]. In VS, vanadium majorly exists in the insoluble spinel, resulting in the direct extraction of vanadium from VS to be difficult. Currently, the primary technology for vanadium extraction from VS is the sodium roasting-water leaching process which has been realized industrialization. In this method, VS is firstly roasted with sodium salts such as Na_2_CO_3_, NaCl or Na_2_SO_4_, among which, Na_2_CO_3_ is commonly used in industrial operations, in a rotary kiln to transform the insoluble spinel to water-soluble sodium vanadates such as NaVO_3_, Na_2_V_2_O_7_ and Na_3_VO_4_ [15]. After that, the roasted VS is water-leached to extract vanadium. As reported [13], vanadium leaching efficiencies of 89.4–98.0% can be achieved by using this process. However, this technology faces several challenges such as high equipment investment, high energy consumption and toxic gases emission due to the roasting process. To address these issues, some other roasting-leaching technologies such as calcium salt roasting-acidic leaching [16], magnesium salt roasting-acidic leaching [17], manganese salt roasting-acidic leaching [18], ammonium salt roasting-acidic leaching [19] and non-salt roasting-acidic or alkaline leaching [20], [21] were developed. Nevertheless, the high equipment cost and high energy consumption generated by the roasting process are still difficult to address. Developing green and efficient approaches for extracting vanadium from the unroasted VS hold substantial economic and environmental significance.

The electro-oxidation method with the advantages of low energy consumption and no toxic gases emission has been applied in extracting vanadium from the unroasted VS. During the electro-oxidation-assisted acidic leaching process, the insoluble trivalent vanadium (V(Ⅲ)) in the spinel can be electro-oxidized to high valence states, promoting vanadium releasing from the unroasted VS [22]. However, the spinel is commonly wrapped by silicates in VS, inhibiting the contact of the spinel with the anodic surface, oxidizing substances, and acidic leaching solution. The leaching efficiency of vanadium is about 70% by using the electro-oxidation-assisted acidic leaching process, which is lower than that of the roasting-leaching methods. Breaking down the silicate-wrapped structure and enlarging the reactive surface area of the vanadium-bearing spinel are essential to enhance vanadium leaching efficiency.

The ultrasound-assisted leaching methods have been widely employed in the extraction of valuable metals from the complex multi-metal minerals [23], [24], [25]. Several researches have reported that the cavitation effects generated by ultrasound treatment of a solution can break down the wrapped structure of mineral particles that significantly improve the leaching kinetics of the target elements [26], [27]. Liu et al. [28] extracted zinc, cadmium, magnesium and copper from the copper-cadmium slag by using the ultrasound-assisted leaching method. The collapse of the cavitation bubbles resulted in a local high temperature and pressure that exposed the reactive surface of the copper-bearing mineral by breaking down the wrapped structure, which reduced the slag particle size from 30 μm to 1 μm and improved solid–liquid contact. Zinc, cadmium, magnesium with the leaching efficiency more than 98% and copper leaching efficiency of 97% were achieved under the conditions of a H_2_SO_4_ concentration of 2  mol L^−1^, a temperature of 40 °C, a reaction time of 2  h, a liquid to solid ratio (L/S) of 3 and an ultrasound power of 300  W. Miao et al. [29] recovered vanadium from low-grade stone coal through ultrasound-assisted leaching technology. They reported that the silicate-wrapped structure of the vanadium-bearing minerals in the low-grade stone coal can be effectively disrupted by ultrasound cavitation, which promoted more efficient contact between sulfuric acid and the vanadium-bearing minerals. Vanadium leaching efficiency of 94.4% was achieved under the optimal conditions of a H_2_SO_4_ concentration of 18 wt%, a temperature of 90 °C, a reaction time of 12  h, a L/S of 1 and an ultrasound power density of 500 W L^−1^. Liu et al. [30] extracted vanadium and molybdenum from vanadium-molybdenum slag by using ultrasound-assisted sodium persulfate leaching method. They reported that the physical fragmentation of minerals caused by ultrasound cavitation improved vanadium and molybdenum leaching kinetics. Under the optimal conditions of a sodium persulfate concentration of 0.3 mol L^−1^. a H_2_SO_4_ concentration of 3 mol L^−1^, a temperature of 90 °C, a reaction time of 2  h, a L/S of 9 and an ultrasound power of 500 W, the application of ultrasonic wave improved the vanadium and molybdenum leaching efficiencies from 86.3% and 93.3% in conventional leaching to 99.5% and 99.8%, respectively.

Recently, the improvement of the ultrasound cavitation effect by inducing electric field into a solution has been demonstrated by researchers [31], [32]. The presence of electric stress in a solution can reduce the surface tension of the cavitation bubble wall, leading to the cavitation bubble collapse becomes easier and more violent [33]. Our previous study showed that the electric field intensified ultrasound cavitation can break down the silicate-wrapped structure of spinel in VS and reduce slag particle size [34]. The vanadium leaching efficiency of 94.64% was achieved by electric field intensified ultrasound leaching, which is 31.08% higher than the ultrasound intensified leaching and 19.66% higher than the electric field intensified leaching. Although electric field combined with ultrasound-assisted leaching has been proven to improve the vanadium leaching efficiency from VS, the fragmentation behavior of VS minerals under the action of electric field intensified ultrasound cavitation has not been fully understood.

In this work, the mineralogical composition and microstructure of VS were determined using X-ray diffraction (XRD), mineral liberation analyzer (MLA) and field emission-scanning electron microscopy (FESEM). The interfacial bonding structure between spinel and silicates was analyzed using high-resolution transmission electron microscopic (HRTEM). Dynamic high-frequency piezoelectric quartz sensor was applied to quantitatively characterize the ultrasonic impact force of ultrasound treatment (UT) and electric field intensified ultrasound treatment (EUT) in water. The relationship between ultrasound working distance (WD) and ultrasonic impact force was revealed. The effects of UT and EUT on the fragmentation of VS minerals were systematically investigated by measuring mineralogical morphology, slag particle size and specific surface area of VS before and after treatments.

## 2 Materials and methods

### 2.1 Materials

The raw vanadium slag (RVS) was provided by Chengde Iron & Steel Group, Hebei Province, China. The chemical composition of this slag was determined in our previous work and shown in Table 1 [34]. The main chemical compositions of this slag are Fe (29.744 wt%), Ti (6.687 wt%), V (6.667 wt%), Cr (3.450 wt%), Mn (4.664 wt%), Si (6.546 wt%), Ca (1.345 wt%), Mg (1.473 wt%), Al (2.032 wt%), and O (36.602 wt%). The bulk RVS was crushed into powders using an ore crusher and sieved with a sieve. The RVS with a particle size (PS) smaller than 300 μm was used for mineralogical and morphology analysis. While, the bulk RVS was also cut into slices with a dimension of 10 mm × 10 mm × 5 mm for UT and EUT tests.

**Table 1:** Chemical composition of the raw vanadium slag (wt.%).

Element	Content	Element	Content	Element	Content
Fe	29.744 ± 2.485	Si	6.546 ± 0.478	P	0.079 ± 0.039
Ti	6.687 ± 1.719	Ca	1.345 ± 0.162	S	0.085 ± 0.017
V	6.667 ± 0.104	Mg	1.473 ± 0.154	Cl	0.101 ± 0.126
Cr	3.450 ± 0.119	Al	2.032 ± 0.044	K	0.115 ± 0.025
Mn	4.664 ± 0.109	Na	0.381 ± 0.082	O	36.602 ± 0.433

### 2.2 UT and EUT tests

#### 2.2.1 UT and EUT of the RVS slice

The prepared RVS slice was placed onto the bottom of a cylindrical reactor with a diameter of 6 cm. To eliminate the influence of chemical reactions on the morphology of the RVS slice, the deionized water (pH = 6.5) without pH adjustment was added into the cylindrical reactor as the medium. This reactor was equipped with two graphite electrodes (25 mm × 25 m m × 3 mm), serving as the anode and cathode, respectively. During the UT and EUT processes, the anode and cathode were placed at a constant distance of 20 mm. The IT6300 (ITECH, China) direct current (DC) power (maximum current = 3 A, maximum voltage = 30 V) was employed as the electric power source. The BHYTS-900 N ultrasonic device (Baihang Ultrasonic Technology Co., Ltd., China) with ultrasound working frequency of 24 kHz and maximum ultrasound power of 1000 W was used as the ultrasonic source. The ultrasonic probe (8 mm in diameter) was vertically placed in the middle of those two graphite electrodes, maintaining working distances (WD) of 0.5–5 cm between the bottom of the ultrasonic probe and the upper surface of the RVS slice. The EUT tests were conducted under the conditions of a current density of 75 mA cm^−2^, a temperature of 25 °C, a reaction time of 2 h, and a ultrasound power of 700 W. For comparing, the UT tests were performed under the same conditions without electric field intensification. Fig. 1 shows the schematic diagrams of UT and EUT of the RVS slice experiments.

**Fig. 1:**
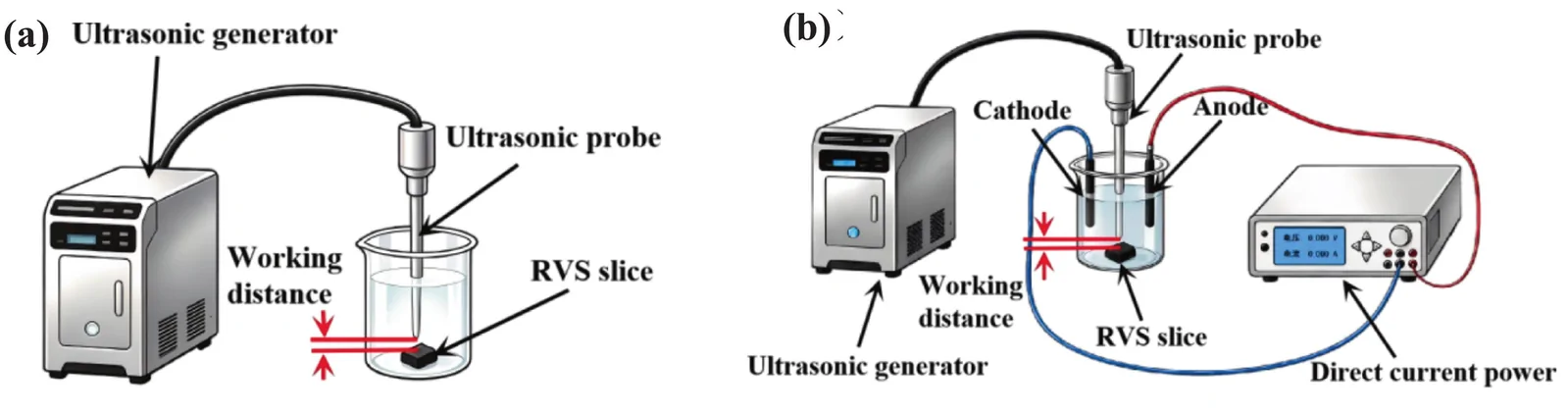
Schematic diagrams of UT and EUT of the RVS slice experiments, (a) UT, (b) EUT.

#### 2.2.2 UT and EUT of the RVS particles with different particle sizes

To investigate the effect of ultrasound cavitation on the fragmentation of the RVS particles with different PSs, the crushed RVS was sieved into five PSs of 40–58 μm, 58–74 μm, 74–120 μm, 120–150 μm, and 150–300 μm using standard sieves. The RVS particles with different PSs were placed in a cylindrical reactor with a diameter of 6 cm. The EUT was performed under the conditions of a current density of 75 mA cm^−2^, a temperature of 25 °C, a reaction time of 2 h, and a ultrasound power of 700 W in deionized water (pH = 6.5). The electrodes, DC power, and ultrasonic device adopted in this experiment was consistent with that described in Section 2.2.1. A magnetic stirrer was applied for stirring the solution at a stirring speed of 600 rpm during the EUT process. For comparing, the UT tests were carried out under the same conditions without electric field intensification.

#### 2.2.3 UT and EUT of the RVS particles in cylindrical reactors with different diameters

To investigate the effect of ultrasound WD on the fragmentation of the RVS particles, the crushed RVS particles with PS < 74 μm was placed in cylindrical reactors with diameters of 8 cm, 6 cm and 4 cm, respectively. The UT and EUT of the RVS particles were performed under the same experimental conditions described in Section 2.2.2.

### 2.3 Dynamic high-frequency piezoelectric quartz sensor measurements

The ZZ500-016–500 N dynamic high-frequency piezoelectric quartz sensor (Shanghai Zhijian Measurement and Control System Co., Ltd., China) with a high response frequency of over 50,000 times per second was employed to in-situ measure the impact force caused by ultrasound cavitation. A piezoelectric quartz sensor with a diameter of 17.5 mm was placed on the bottom of a cylindrical reactor. The 8 mm-ultrasonic probe was vertically placed in the middle of those two graphite electrodes, maintaining WD of 0.5–10 cm. By processing the signals emitted by the piezoelectric quartz sensor via a computer, the ultrasonic impact force of EUT of x-axis and y-axis were determined. The UT tests were conducted under the same conditions without electric field intensification. Both the EUT and UT were carried out under the same experimental conditions described in Section 2.2.1. Fig. 2 shows the schematic diagrams of the dynamic high-frequency piezoelectric quartz sensor measurements under the action of UT and EUT.

**Fig. 2:**
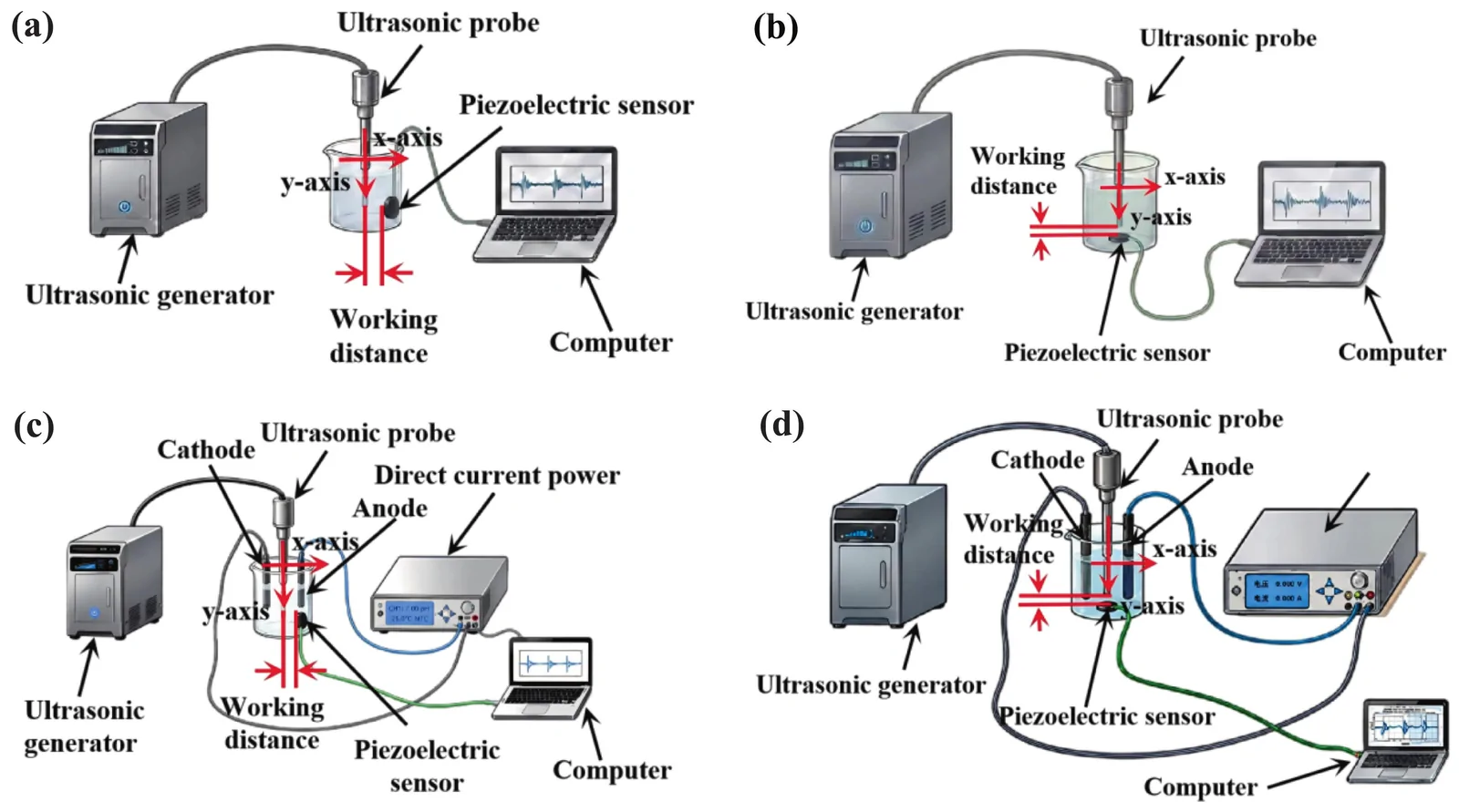
Schematic diagrams of the dynamic high-frequency piezoelectric quartz sensor measurements under the action of UT and EUT, (a) UT on x-axis, (b) UT on y-axis, (c) EUT on x-axis, (d) EUT on y-axis.

### 2.4 Characterizations

The mineralogical compositions of the RVS were detected by XRD (Brucker D8 Advance, Brucker, Germany) with Cu-Kα radiation conducted at 40 kV and 100 mA. The RVS was scanned in the 2θ range from 10° to 90° using a scanning rate of 10° 2θ min^−1^. A TIMA-X MLA (TESCAN ORSAY HOLDING, Czechia) was employed to further identify the mineralogical content and distribution of the RVS. The morphology of the RVS before and after UT and EUT were characterized by using SU8020 FESEM coupled to an energy dispersive spectrometer (EDS). The transmission electron microscopic (TEM) images and HRTEM images were obtained on FEI Tecnai G2 F20 transmission electron microscope (FEI, USA) to investigate the microstructure of the RVS. Mastersizer 2000 laser particle size analyzer (Malvern, UK) and ASAP 2460 (Mcmurretik Instrument, USA) surface area analyzer were employed to characterize the PS distribution and specific surface area (SSA) of the RVS before and after UT and EUT, respectively.

## 3 Results and discussion

### 3.1 Mineralogical composition and microstructure of the RVS

Fig. 3(a) shows the XRD patterns of the RVS. The RVS mainly composed of spinel, fayalite and hematite. The MLA analysis results shown in Fig. 3(b) and Table 2 imply that the weight contents of spinel and fayalite were 68.04 wt% and 22.44 wt%, respectively, accounting for 90.48 wt% of the total mass of the RVS. Similar results were obtained by Li et al. [35] and Wang et al. [36]. They also indicated that the spinel and fayalite are the major minerals in the RVS. Besides spinel and fayalite, the ferro-aluminotschermakite and hematite with weight contents of 2.30 wt% and 4.01 wt%, respectively, were detected in the RVS through MLA analysis. In addition, the RVS also contained a small amount of ankerite, metallic Fe, garnet, calcite, hornblende, pseudobrookite, periclase, quartz, etc.

**Fig. 3:**
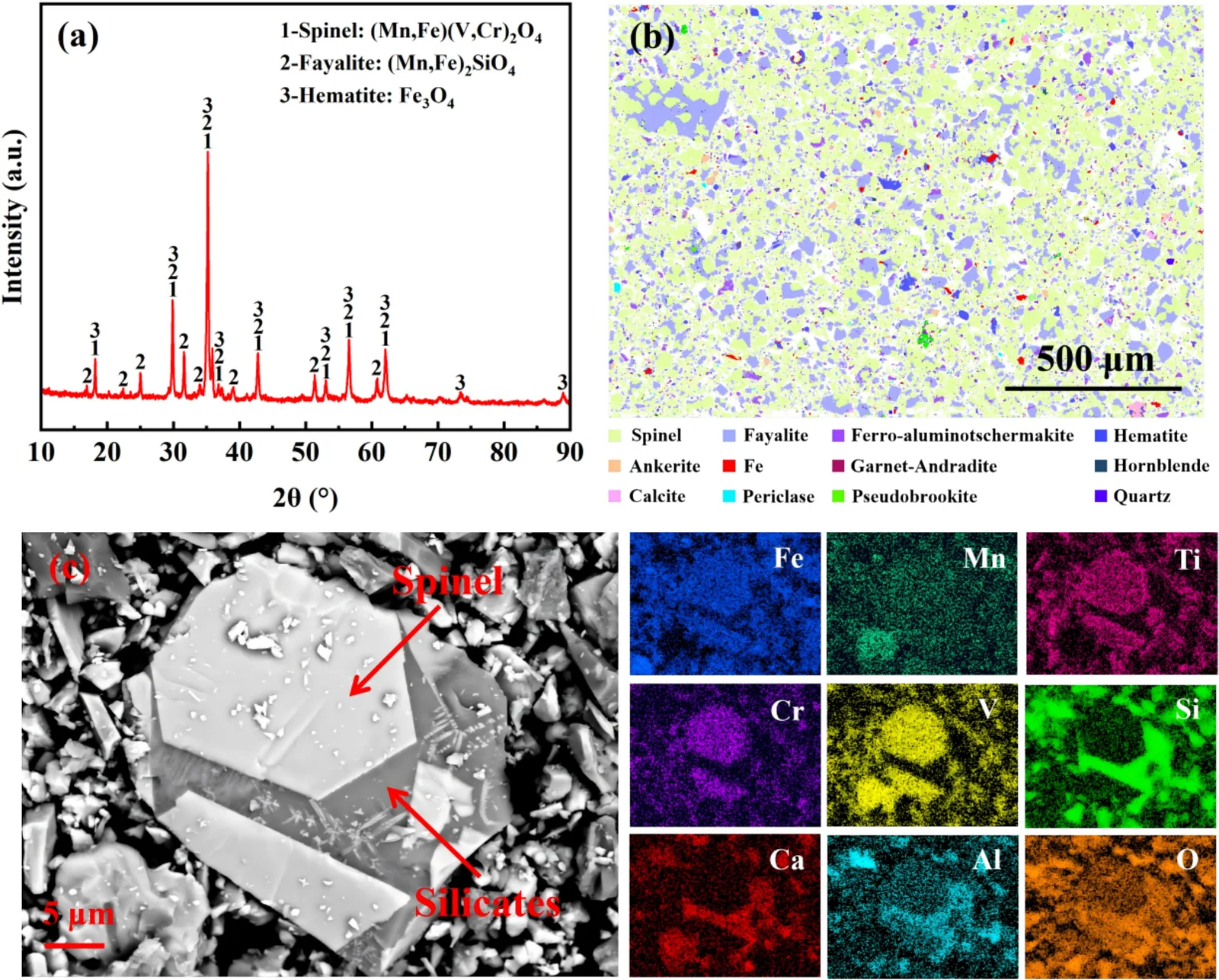
Mineralogical composition and morphology of the RVS, (a) XRD patterns, (b) MLA analysis, (c) FESEM-EDS analysis.

**Table 2:** Mineralogical content of the RVS determined by MLA analysis, in wt.%.

Primary minerals	Content
Spinel	68.04
Fayalite	22.44
Hematite	4.01
Ferro-aluminotschermakite	2.30
Ankerite	0.58
Metallic Fe	0.52
Garnet-Andradite	0.44
Calcite	0.40
Pseudobrookite	0.39
Hornblende	0.33
Others	0.55

Fig. 3(c) exhibits that the FESEM image of the RVS involved the light region and the dark region. The main elements in the light region were Fe, Mn, V, Cr, and O, corresponding to the spinel of the RVS. While, the major elements in the dark region were Fe, Mn, Ca, Si, Al, and O, which mainly corresponded to silicates such as fayalite and ferro-aluminotschermakite. It can be clearly observed that the spinel was wrapped by silicates in the RVS.

To further investigate the microstructure of the RVS, TEM analysis was performed. Fig. 4(a)–(c) show that the elemental V, Cr, Mn and Fe mainly existed in I_1_ region, indicating that the mineral in I_1_ region was spinel. While, the elemental Si, Mn and Fe majorly existed in I_2_ region. This suggests that the mineral in I_2_ region was fayalite. As shown in Fig. 4(d), the crystal interplanar space measured in I_1_ region was 2.539 Å. According to the International Centre for Diffraction Data (ICDD) database, the crystal interplanar spaces of vanadium-iron spinel (FeV_2_O_4_) (3, 1, 1), chromium-iron spinel (FeCr_2_O_4_) (3, 1, 1), vanadium-manganese spinel (MnV_2_O_4_) (3, 1, 1) and chromium-manganese spinel (MnCr_2_O_4_) (3, 1, 1) are 2.549 Å (ICDD: 04-006-0430), 2.530 Å (ICDD: 97-004-4526), 2.560 Å (ICDD: 04-006-5400) and 2.544 Å (ICDD: 04-006-2565), respectively. The detected crystal interplanar space in I_1_ region was higher than that of FeCr_2_O_4_ spinel, but lower than that of FeV_2_O_4_ spinel, MnV_2_O_4_ spinel and MnCr_2_O_4_ spinel. This indicates that the spinel detected in the RVS is a solid solution. The crystal interplanar space measured in I_2_ region was 5.310 Å, which corresponded to the fayalite (0, 2, 0) (ICDD: 00-012-0220). It is noteworthy that an obvious change in crystal interplanar space can be observed at the grain boundaries (I_3_ region) between the spinel and fayalite. This indicates that these two phases is mechanical connection. Under the action of ultrasound cavitation, the intergranular mechanical connection structure is likely to be destroyed, which would improve the reactive surface area of the vanadium-bearing spinel.

**Fig. 4:**
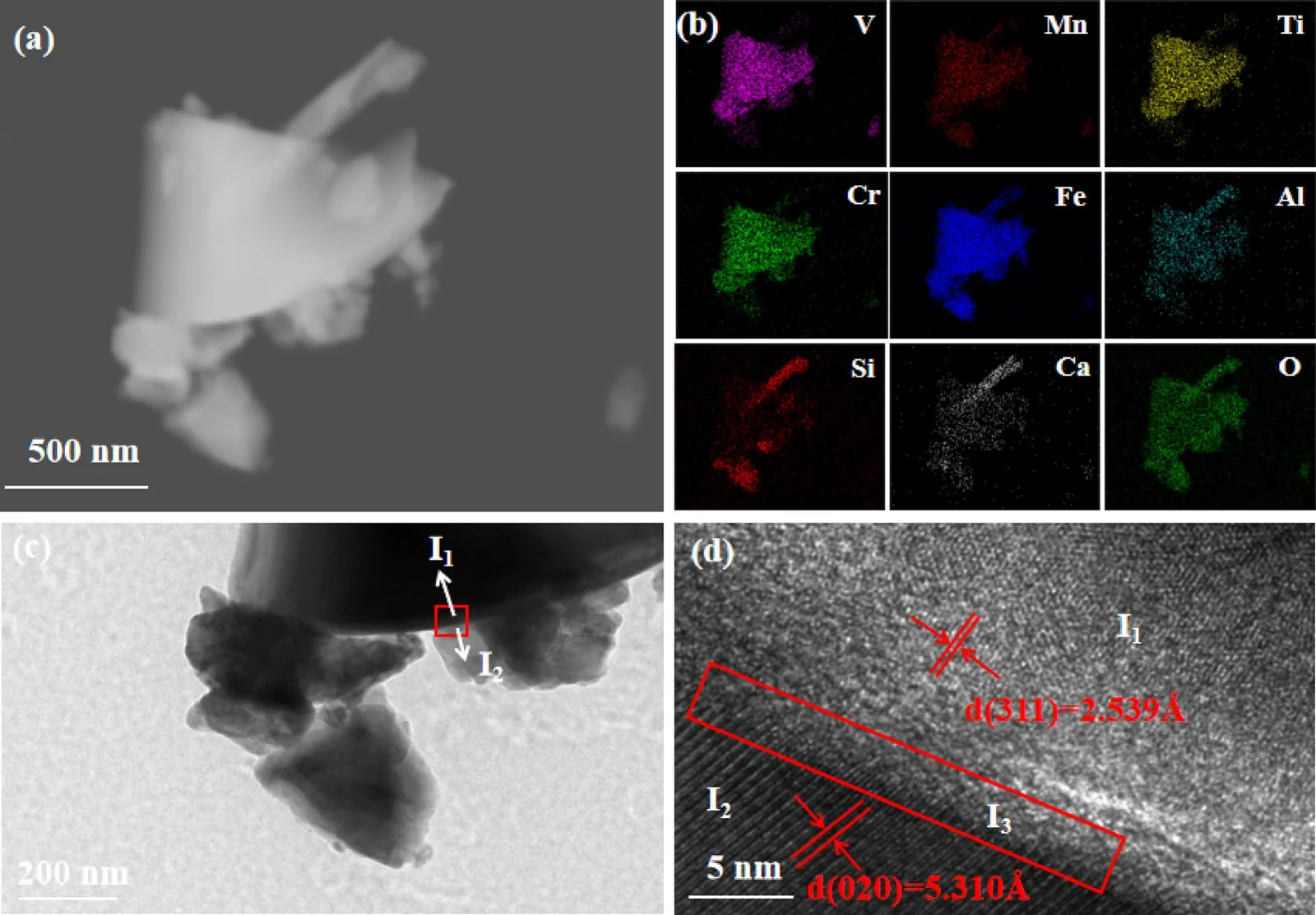
TEM analysis of the RVS, (a) TEM image of the RVS at a low magnification, (b) combined elemental mapping in (a), (c) TEM image of the RVS at a high magnification, (d) HRTEM image of the selected area.

### 3.2 Effects of ultrasound cavitation on morphology of the RVS

The prepared RVS slice was placed below the ultrasonic probe. By adjusting the WD between the RVS slice and ultrasonic probe, the effects of ultrasound cavitation on morphology of the RVS were investigated. Fig. 5 shows the evolution of RVS slice surface morphology as a function of ultrasound WD under UT. As shown in Fig. 5(a)–(d), the morphology of the RVS slice almost not changed when the ultrasound WD was higher than 2 cm. The broken of the dark region of the silicates was observed when the ultrasound WD was 1 cm (Fig. 5(e)). The further reduction in ultrasound WD to 0.5 cm intensified mineral damage of the RVS (Fig. 5(f)). The cracks were detected both on the surface of the spinel and silicates and at the boundaries between these phases. This indicates that the RVS minerals can be destroyed at a sufficiently small ultrasound WD under UT.

**Fig. 5:**
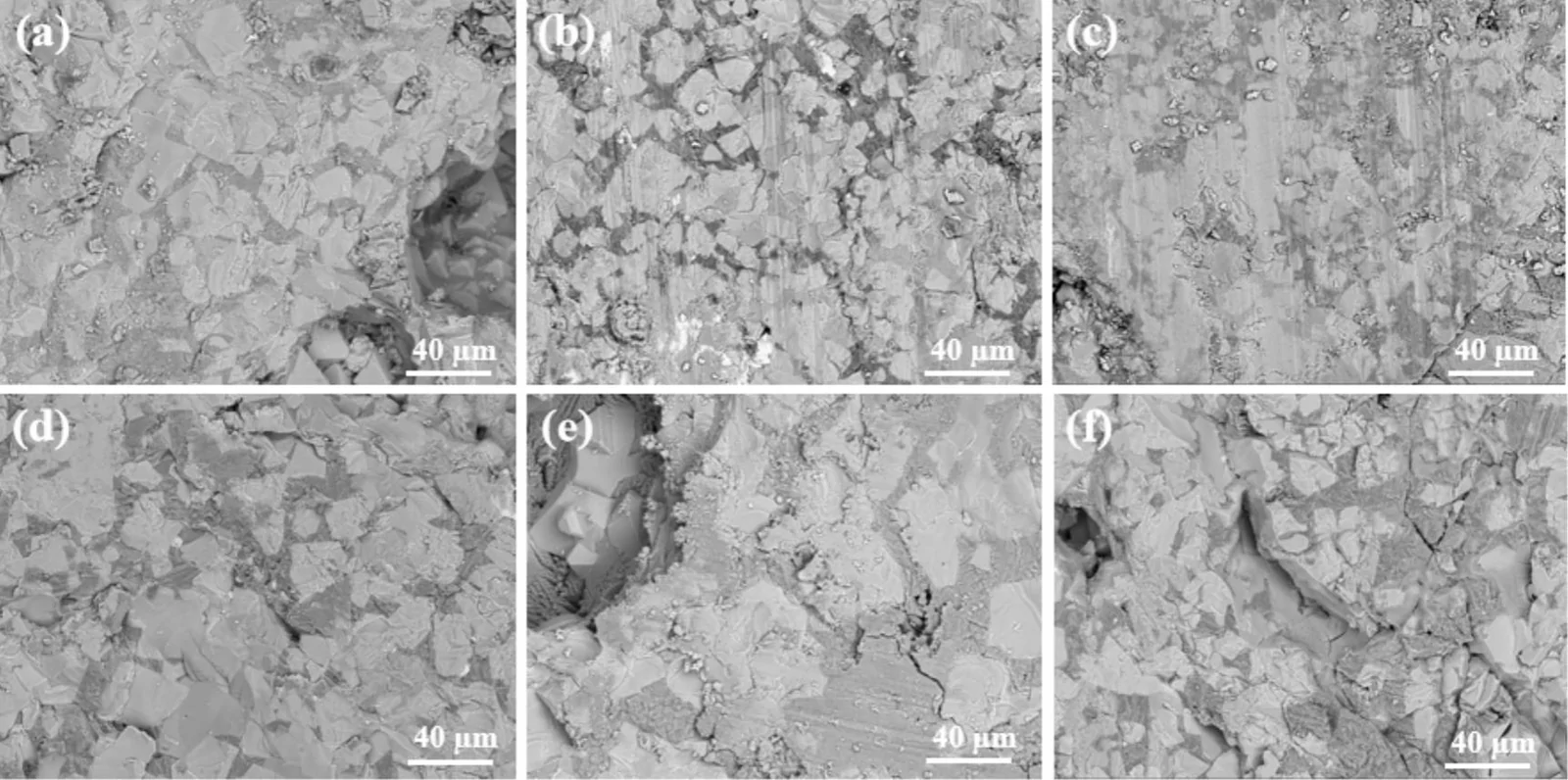
Evolution of the RVS slice surface morphology as a function of ultrasound WD under UT, (a) WD = 5 cm, (b) WD = 4 cm, (c) WD = 3 cm, (d) WD = 2 cm, (e) WD = 1 cm, (f) WD = 0.5 cm.

The cavitation bubbles commonly undergo formation, growth and collapse processes when ultrasound waves exist in a solution [37]. In a solid–liquid system, both asymmetric and symmetric collapses of the cavitation bubbles can be occurred, depending on the distance between the solid particles and the generated cavitation bubbles. When the solid particles are very close to the cavitation bubbles, the asymmetric collapse of the cavitation bubbles would occur that forms powerful liquid jets [38], [39]. As reported, the collapse of the cavitation bubbles produces a micro-environment with localized high temperatures greater than 5000 K and high pressures greater than 500 atm, which generates strongly mechanical effect [40]. The formed microjets would directly act on the surface of the RVS slice, inducing the broken of the minerals.

Fig. 6 shows the evolution of the RVS slice surface morphology as a function of ultrasound WD under EUT. As shown in Fig. 6(a)–(d), the morphology of the RVS slice also not changed when the WD of ultrasound was higher than 2 cm. The damage of the RVS minerals can be obviously observed when the ultrasound WD was 1 cm (Fig. 6(e)). Comparing with UT, the extent of mineral damage was greater when the RVS slice was treated by electric field intensified ultrasound. This can be attributed to that the presence of electric field decreases the surface tension of the generated cavitation bubbles wall, resulting in the maximum bubble-radius to be larger during the rarefaction cycle and the minimum bubble-radius to be smaller during the compression cycle. The gas pressure and temperature in the center of the cavitation bubbles significantly enhanced when exposing to electric field intensified ultrasound environment instead of ultrasound alone [33]. In addition, with the electric field intensification, the cavitation threshold for bubbles also reduced. The decrease in surface tension of bubbles wall and the reduction of cavitation threshold by inducing electric field make bubble cavitation occur more violently and easily, which strengthened the mineral fragmentation, compared to that without electric field intensification. As shown in Fig. 6(f), the further reduction in ultrasound WD to 0.5 cm improved the broken degree of the minerals. Due to the extensive fragmentation of minerals, the surface of the RVS slice became rough. It can be also observed that the broken degree of the silicates (dark region) was greater than that of the spinel (light region) of the RVS. The broken degree of mineral is correlated to its mechanical properties and the ultrasonic impact force. The RVS minerals would be fragmented when the ultrasonic impact force was stronger enough [41]. The differences in the mechanical properties of hardness, elastic modulus, fracture toughness, etc. between the silicates and the spinel led to the selective mineral fragmentation. As reported in the literature [42], [43], [44], [45], the mechanical properties such as hardness, elastic modulus, fracture toughness of the fayalite-type mineral, which is the major silicate in the RVS, were much lower than that of the spinel-type mineral. This may be the main reason for the greater broken degree of the silicates than that of the spinel during EUT.

**Fig. 6:**
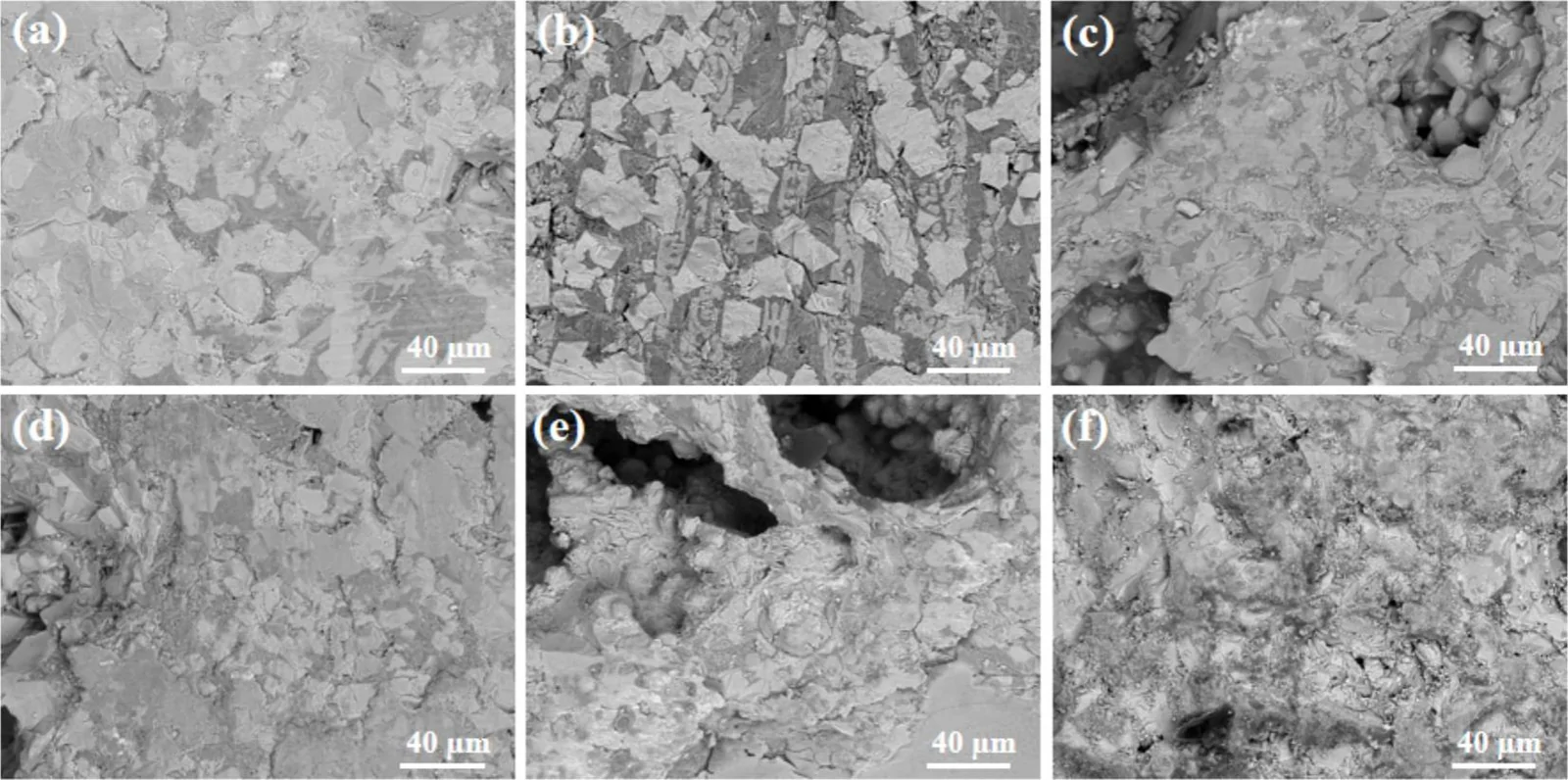
Evolution of the RVS slice surface morphology as a function of ultrasound WD under EUT, (a) WD = 5 cm, (b) WD = 4 cm, (c) WD = 3 cm, (d) WD = 2 cm, (e) WD = 1 cm, (f) WD = 0.5 cm.

Piezoelectric sensor measurements were performed to quantitatively characterize the effect of ultrasound WD on the impact force of ultrasound. As shown in Fig. 7, the rapid collapse of cavitation bubbles within 0.004–0.006 s induced impact force on the surface of the quartz sensor. With the decrease of y-axis ultrasound WD from 5 cm to 0.5 cm, the y-axis ultrasonic impact force enhanced from 5.72 N to 25.21 N. It is noteworthy that the improvement of the ultrasonic impact force was insignificant with the decrease in ultrasound WD from 5 cm to 3 cm. The obvious increase in the ultrasonic impact force can be found when the ultrasound WD was lower than 3 cm. This indicates that the asymmetric collapse of cavitation bubbles can be effectively intensified by reducing the bubble-solid surface distance within 0–3 cm.

**Fig. 7:**
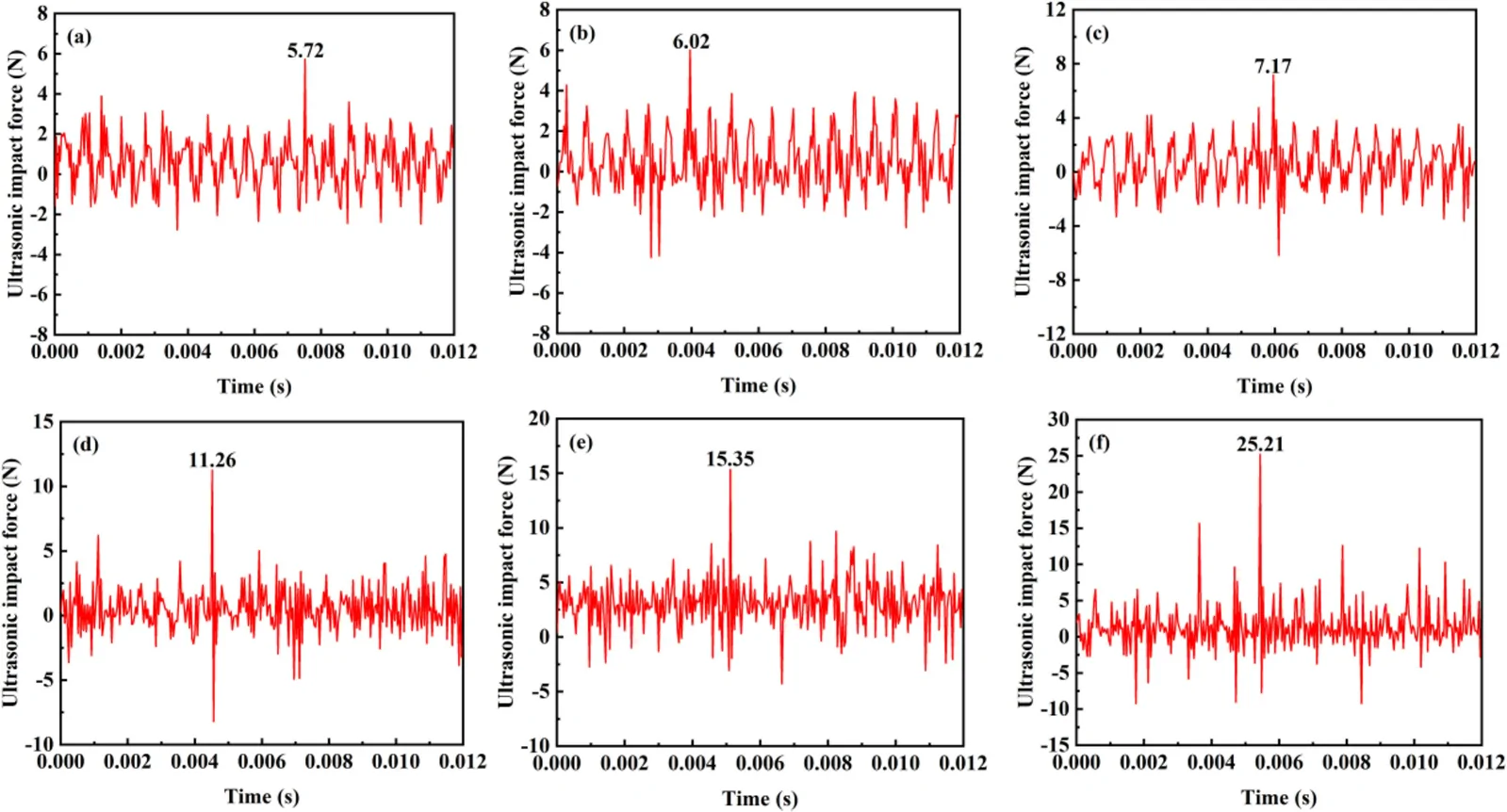
Evolution of y-axis ultrasonic impact force as a function of y-axis ultrasound WD under UT, (a) WD = 5 cm, (b) WD = 4 cm, (c) WD = 3 cm, (d) WD = 2 cm, (e) WD = 1 cm, (f) WD = 0.5 cm.

Fig. 8 shows the evolution of y-axis ultrasonic impact force as a function of y-axis ultrasound WD under EUT. With the decrease in y-axis ultrasound WD from 5 cm to 0.5 cm, the y-axis ultrasonic impact force increased from 6.66 N to 38.65 N. The significant enhancement of ultrasonic impact force was also observed when the ultrasound WD was lower than 3 cm under EUT. Inducing electric field into ultrasound field improved the ultrasonic impact force. At the y-axis ultrasound WD of 0.5 cm, the y-axis ultrasonic impact force of the UT alone increased by 53.31% through inducing electric field. It is noteworthy that the ultrasonic impact force rose rapidly and then decayed within the time window of 0.004–0.006 s as shown in Figs. 7 and 8. The ultrasonic impact force value measured by the piezoelectric sensor includes multiple events such as cavitation-induced microjets, shock wave, pressure fluctuations, mechanical vibration, etc. [41]. The ultrasonic impact force generated by cavitation bubble collapse is much stronger than other effects. The cavitation bubble collapse is instantaneous, while the shock wave, pressure fluctuations and mechanical vibration are sustained. Therefore, the measured ultrasonic impact force rose rapidly and then decayed.

**Fig. 8:**
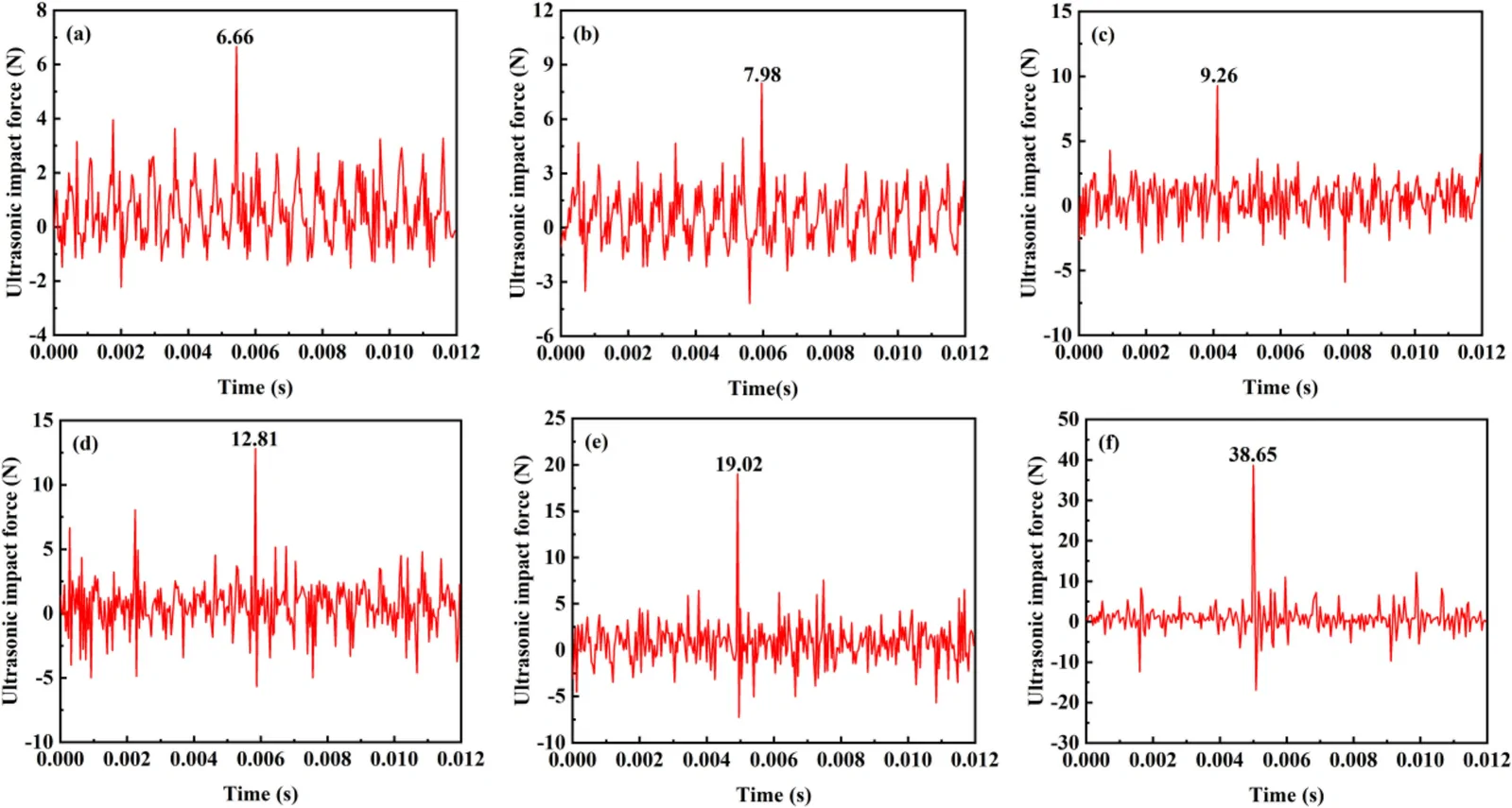
Evolution of y-axis ultrasonic impact force as a function of y-axis ultrasound WD under EUT, (a) WD = 5 cm, (b) WD = 4 cm, (c) WD = 3 cm, (d) WD = 2 cm, (e) WD = 1 cm, (f) WD = 0.5 cm.

Through fitting the results determined by piezoelectric sensor measurements, the relationship curves between ultrasonic impact force and ultrasound WD were obtained. As shown in Fig. 9, under the action of ultrasound alone and electric field intensified ultrasound, the variations of ultrasonic impact force on the x-axis and y-axis with ultrasound WD all conformed to the natural exponential functional relationship, as shown in equation (1). The correlation coefficient (R^2^) values of these four curves were all higher than 0.96.

(1) $$\text{y = A}{\text{e}}^{\text{(- [GSLL]x}\text{)}}\;\text{+ C}$$

where, y is the ultrasonic impact force, A is the pre-exponential factor, λ is the decay exponent.

**Fig. 9:**
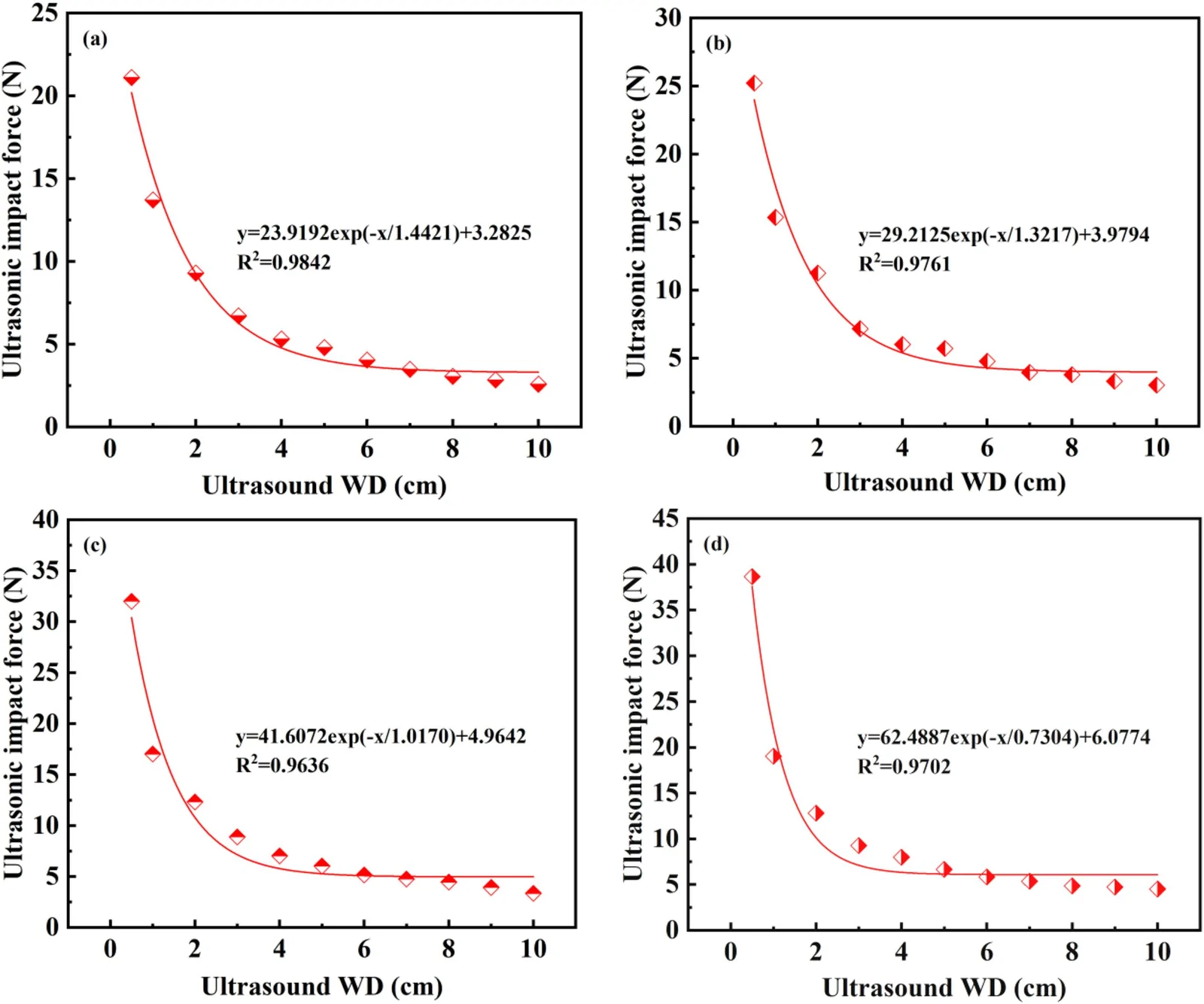
Relationship between ultrasound WD and ultrasonic impact force under UT and EUT, (a) x-axis ultrasonic impact force vs. x-axis ultrasound WD under UT, (b) y-axis ultrasonic impact force vs. y-axis ultrasound WD under UT, (c) x-axis ultrasonic impact force vs. x-axis ultrasound WD under EUT, (d) y-axis ultrasonic impact force vs. y-axis ultrasound WD under EUT.

As for the UT, the relationship between ultrasonic impact force and ultrasound WD on x-axis and y-axis can be described as Eqs. (2), (3), respectively.

(2) $$\text{Ultrasonic impact force = 23.9192}{\text{e}}^{\text{(-}\;\frac{\text{ultrasound WD}}{\text{1.4421}}\text{)}}\;\text{+ 3.2825}$$

(3) $$\text{Ultrasonic impact force = 29.2125}{\text{e}}^{\text{(-}\;\frac{\text{ultrasound WD}}{\text{1.3217}}\text{)}}\;\text{+ 3.9794}$$

While, for the EUT, the relationship between ultrasonic impact force and ultrasound WD on x-axis and y-axis can be described as Eqs. (4), (5), respectively.

(4) $$\text{Ultrasonic impact force = 41.6072}{\text{e}}^{\text{(-}\;\frac{\text{ultrasound WD}}{\text{1.0170}}\text{)}}\;\text{+ 4.9642}$$

(5) $$\text{Ultrasonic impact force = 62.4887}{\text{e}}^{\text{(-}\;\frac{\text{ultrasound WD}}{0.7304}\text{)}}\;\text{+ 6.0774}$$

The pre-exponential factor values of Eqs. (4), (5) were higher than that of Eqs. (2), (3). This indicates that the ultrasonic impact force can be improved by inducing electric field into ultrasound field both on x-axis and y-axis, which is beneficial to the fragmentation of minerals. Eqs. (2)–(5) also show that the pre-exponential factor values on the y-axis were higher than that on x-axis. The ultrasonic impact force acting on the y-axis was stronger than that on the x-axis. This can be attributed to that under the effect of acoustic streaming, most of the cavitation bubbles mainly diffused along the y-axis direction of the ultrasonic probe, while fewer cavitation bubbles diffused along the x-axis. The collapse of the cavitation bubbles is related to the acoustic pressure. Ultrasonic wave generates alternating positive and negative pressure cycles as it propagates in the solution. During the negative half-cycle of the ultrasonic wave, the cavitation nuclei formed in the solution expand into bubbles, which are then compressed during the positive half-cycle until they reach the maximum size where they collapse [40]. The larger peak acoustic pressure of the y-axis direction of the ultrasonic probe led to the greater collapse of the cavitation bubbles, producing stronger ultrasound impact force acting on the y-axis direction than that on the x-axis direction [46], [47]. It is noteworthy that the EUT with decay exponent values of 0.9833 on x-axis and 1.3691 on y-axis were larger than that of UT (0.6934 on x-axis and 0.7566 on y-axis). Although inducing electric field can improve ultrasonic impact force, the impact force decayed more rapidly with increasing ultrasound WD, compared to ultrasound alone. To guarantee the fragmentation effect on the RVS minerals, the equipment used for treating the RVS via EUT should ensure a sufficiently small reaction space.

### 3.3 Effects of ultrasound cavitation on fragmentation of the RVS particles

In industrial practice, the bulk RVS is commonly subjected to crushing and sieving first, and the obtained RVS particles are subsequently used for vanadium extraction. The effects of ultrasound cavitation on fragmentation of the RVS particles with different PSs were investigated in this work. As shown in Fig. 10(a), (b), (c), (f), (g), (h), (k), (l) and (m), the RVS particles with PS higher than 74 μm can not be well fragmented both under UT and EUT. Due to the impact effect of the cavitation bubbles, the surface of slag particles became rough. The damage of the silicates is significantly stronger than that of the spinel. The reaction surface area of the spinel of RVS can be enlarged by breaking silicates through UT and EUT. The significant fragmentation of the RVS particles through UT and EUT can be observed when slag PS was lower than 74 μm (Fig. 10(d), (e), (i), (j), (n) and (o)). After UT and EUT, the slag particles became finer. The amounts of slag particles with low PS increased.

**Fig. 10:**
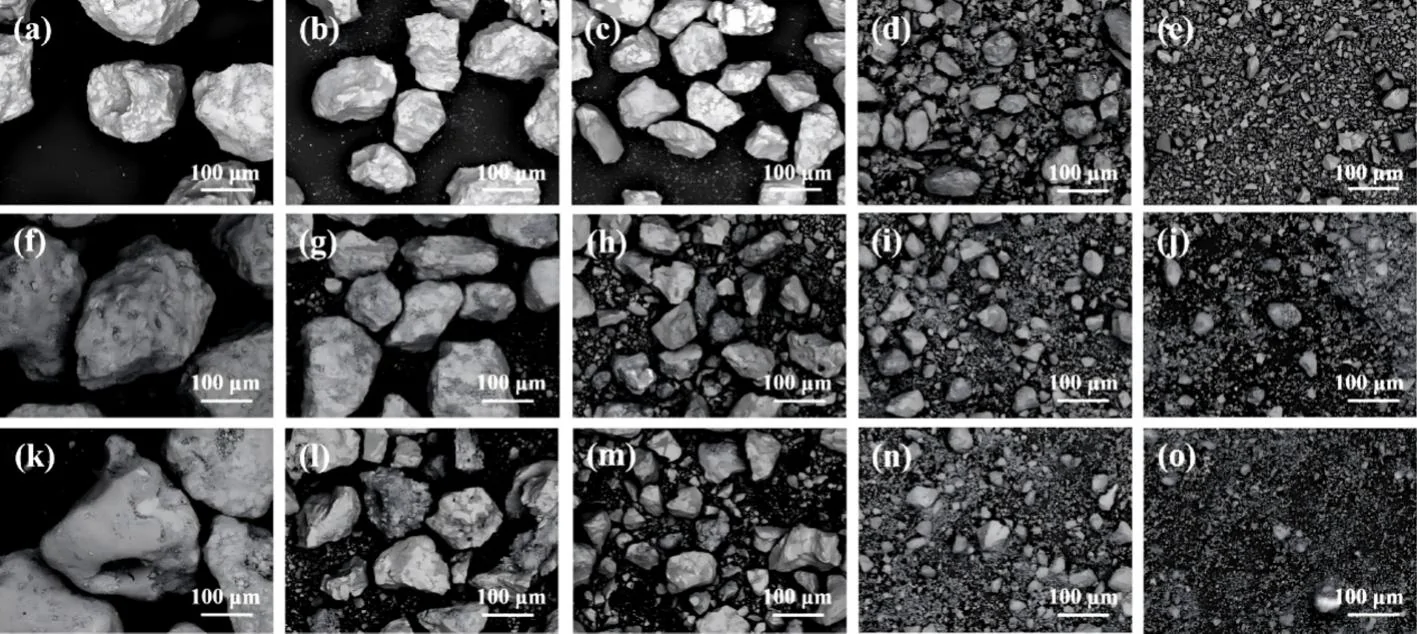
Morphology of the RVS particles with different PSs before and after UT and EUT, (a)-(e) RVS before UT and EUT, (f)-(j) RVS after UT, (k)-(o) RVS after EUT.

PS and Brunauer-Emmett-Teller (BET) measurements were performed to further investigate the fragmentation behavior of the RVS particles through UT and EUT. As shown in Fig. 11(a), the D_50_ value of the RVS particles with 150 μm < PS < 300 μm decreased from 234 μm to 206 μm after UT, while it reduced from 234 μm to 166 μm after EUT. The D_50_ value of the RVS particles with 120 μm < PS < 150 μm decreased from 140 μm to 133 μm and 104 μm after UT and EUT, respectively. As for the VS particles with 74 μm < PS < 120 μm, the D_50_ value decreased from 103 μm to 93.3 μm and 81.8 μm after UT and EUT, respectively. This indicates that both the ultrasound cavitation and the electric field intensified ultrasound cavitation had little effect on the fragmentation of the large-sized RVS particles. The PS of the treated RVS particles was still large. The significant decrease in PS was detected from the RVS with 40 μm < PS < 74 μm through UT and EUT. For the slag with 58 μm < PS < 74 μm, the D_50_ value reduced from 68.8 μm to 21.4 μm and 18.8 μm after UT and EUT, respectively, representing decreased of 68.9% and 72.7%. While, for the slag with 40 μm < PS < 58 μm, the D_50_ value reduced from 54.1 μm to 5.47 μm and 4.73 μm after UT and EUT, respectively, decreasing of 89.9% and 91.3%. The ultrasound cavitation can effectively fragment the fine-grained RVS particles and reduce their PS. The effect of ultrasound cavitation on reducing slag PS can be strengthened by inducing electric field into ultrasound field.

**Fig. 11:**
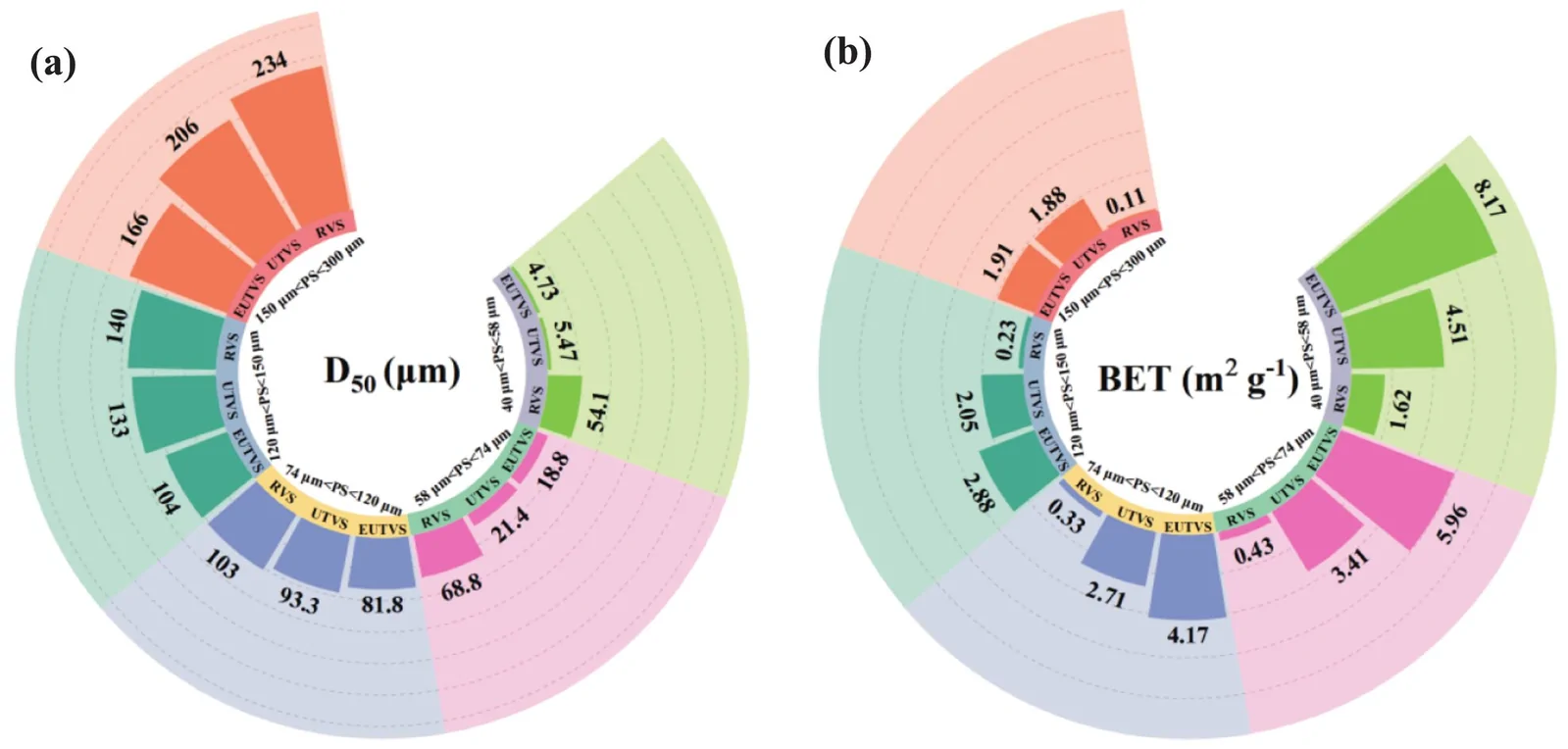
PS distribution and SSA of the RVS particles with different PSs before and after UT and EUT, (a) variation of PS, (b) variation of SSA.

Fig. 11(b) implies that the SSA of the RVS particles with different PSs can be significantly enhanced via UT and EUT. The SSA value of the RVS particles with 150 μm < PS < 300 μm increased from 0.11 m^2^ g^−1^ to 1.88 m^2^ g^−1^ after UT, and it enhanced from 0.11 m^2^ g^−1^ to 1.91 m^2^ g^−1^ after EUT. While, for the RVS with 120 μm < PS < 150 μm, the SSA value improved from 0.23 m^2^ g^−1^ to 2.05 m^2^ g^−1^ and 2.88 m^2^ g^−1^ after UT and EUT, respectively. As mentioned above, the UT and EUT had slight effect on the reduction of large-sized slag PS. Therefore, the increase in SSA of the RVS with PS higher than 120 μm can be mainly attributed to the improvement of the mineral surface roughness due to the damage of silicates. The obtained results also indicates that inducing electric field into ultrasound field has no significant enhancement of the SSA of the large-sized RVS particles, compared to ultrasound alone. As for the RVS with 58 μm < PS < 74 μm, the SSA value improved from 0.43 m^2^ g^−1^ to 3.41 m^2^ g^−1^ and 5.96 m^2^ g^−1^ after UT and EUT, respectively. While, for the RVS with 40 μm < PS < 58 μm, the SSA value increased from 1.62 m^2^ g^−1^ to 4.51 m^2^ g^−1^ and 8.17 m^2^ g^−1^ after UT and EUT, respectively. The improvement of the SSA of the fine-grained RVS particles is attributed to both the increase in mineral surface roughness and the significant reduction in PS. The EUT exhibited a markedly stronger promoting effect on the increase of SSA of the RVS particles than that of UT.

Vanadium leaching from the RVS is a solid–liquid reaction [48], [49], [50]. When the leachant is treated by ultrasonic wave, the collapse of the cavitation bubbles produces microjets [38], [51]. Under the impact of the generated microjets, the silicates of the RVS would be effectively damaged and the slag PS would be reduced, which are beneficial to exposing the reactive surface of the encapsulated vanadium-bearing spinel. The ultrasound cavitation can accelerate the solid–liquid reaction between the leachant and the vanadium-bearing spinel, which would shorten the leaching time and improve the vanadium leaching efficiency [28], [34], [52]. Inducing electric field into ultrasound field makes bubble cavitation occur more violently and easily and enhances the impact force on minerals, which is expected to further improve the vanadium leaching rate. As described above, the reduction of PS and the improvement of SSA was significant when the electric field intensified ultrasound technology was applied to treat the RVS with PS below 74 μm. Therefore, the RVS with PS smaller than 74 μm is recommended to be selected to guarantee high vanadium leaching efficiency using EUT.

To explore the effect of ultrasound WD on the fragmentation of the RVS minerals, the RVS with PS smaller than 74 μm (D_50_ = 26.8 μm, SSA = 0.51 m^2^ g^−1^) was UT and EUT in cylindrical reactors with diameters of 8 cm, 6 cm and 4 cm, respectively. Fig. 12(a) shows that only a small part of the silicates of the RVS can be destroyed under UT in the cylindrical reactor with a diameter of 8 cm. With the reduction in cylindrical reactor diameter, the extent of mineral damage was enhanced, accompanied by increased surface roughness of minerals (Fig. 12(b) and (c)). Notably, when only UT was applied, severe damage to the spinel cannot be observed for the RVS processed in the 4 cm-diameter cylindrical reactor. As shown in Fig. 12(d)–(f), the extent of mineral damage was enhanced with the reduction in cylindrical reactor diameter under EUT. Inducing electric field into ultrasound field intensified the ultrasound cavitation impact. In cylindrical reactors with diameters of 6 cm and 4 cm, the broken of spinel can be observed under EUT due to the strengthened ultrasound cavitation.

**Fig. 12:**
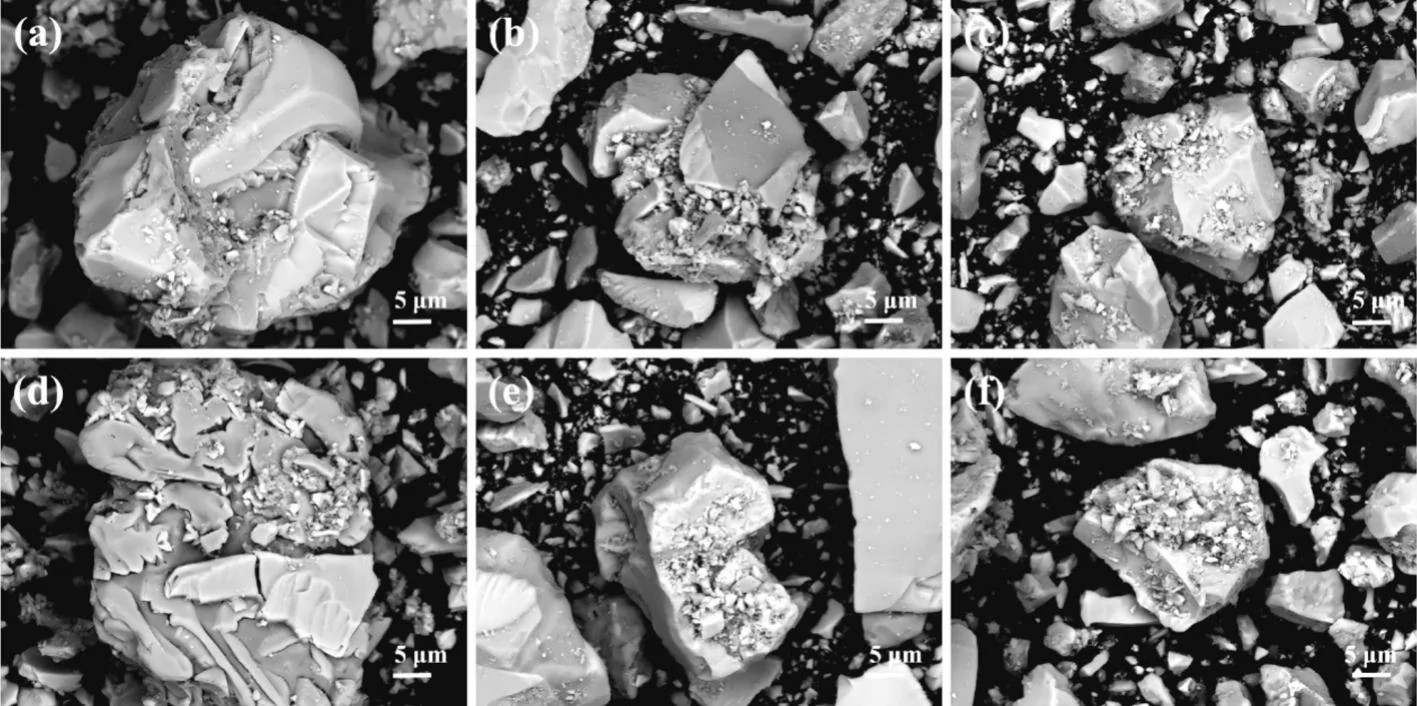
Morphology of the RVS particles after UT and EUT in cylindrical reactors with different diameters, (a) UT in a cylindrical reactor with a diameter of 8 cm, (b) UT in a cylindrical reactor with a diameter of 6 cm, (c) UT in a cylindrical reactor with a diameter of 4 cm, (d) EUT in a cylindrical reactor with a diameter of 8 cm, (e) EUT in a cylindrical reactor with a diameter of 6 cm, (f) EUT in a cylindrical reactor with a diameter of 4 cm.

Fig. 13(a) and (b) show that the D_50_ value of the RVS decreased from 26.8 μm to 21.1 μm and 19.6 μm under UT in cylindrical reactors with diameters of 8 cm and 6 cm, respectively. As mentioned above, the improvement of ultrasonic impact force was slight with the decrease in ultrasonic WD from 4 cm to 3 cm. The effect of ultrasound cavitation on the reduction in PS of the RVS particles was near in cylindrical reactors with diameters of 8 cm and 6 cm under UT alone. As shown in Fig. 13(c), the further decrease in cylindrical reactor diameter to 4 cm reduced the D_50_ value of the RVS from 26.8 μm to 12.1 μm. This can be attributed to that the ultrasonic impact force was significantly enhanced when decreasing ultrasound WD to the values lower than 3 cm. The RVS particles with the D_50_ value of 26.8 μm can be effectively fragmented into smaller-sized grains under the action of ultrasound cavitation in cylindrical reactors with diameters lower than 4 cm. Fig. 13(d) shows that the UT and EUT show no obvious difference in reducing RVS PS when operating in the cylindrical reactor with a diameter of 8 cm. Decreasing the cylindrical reactor diameters to 6 cm and 4 cm further reduced the PS to 16.3 μm and 10.4 μm, respectively, under the action of EUT (Fig. 13(e) and (f)). The enhancement effect of electric field on ultrasonic cavitation in cylindrical reactors with diameters of 6 cm and 4 cm was superior to that in the 8 cm-diameter cylindrical reactor.

**Fig. 13:**
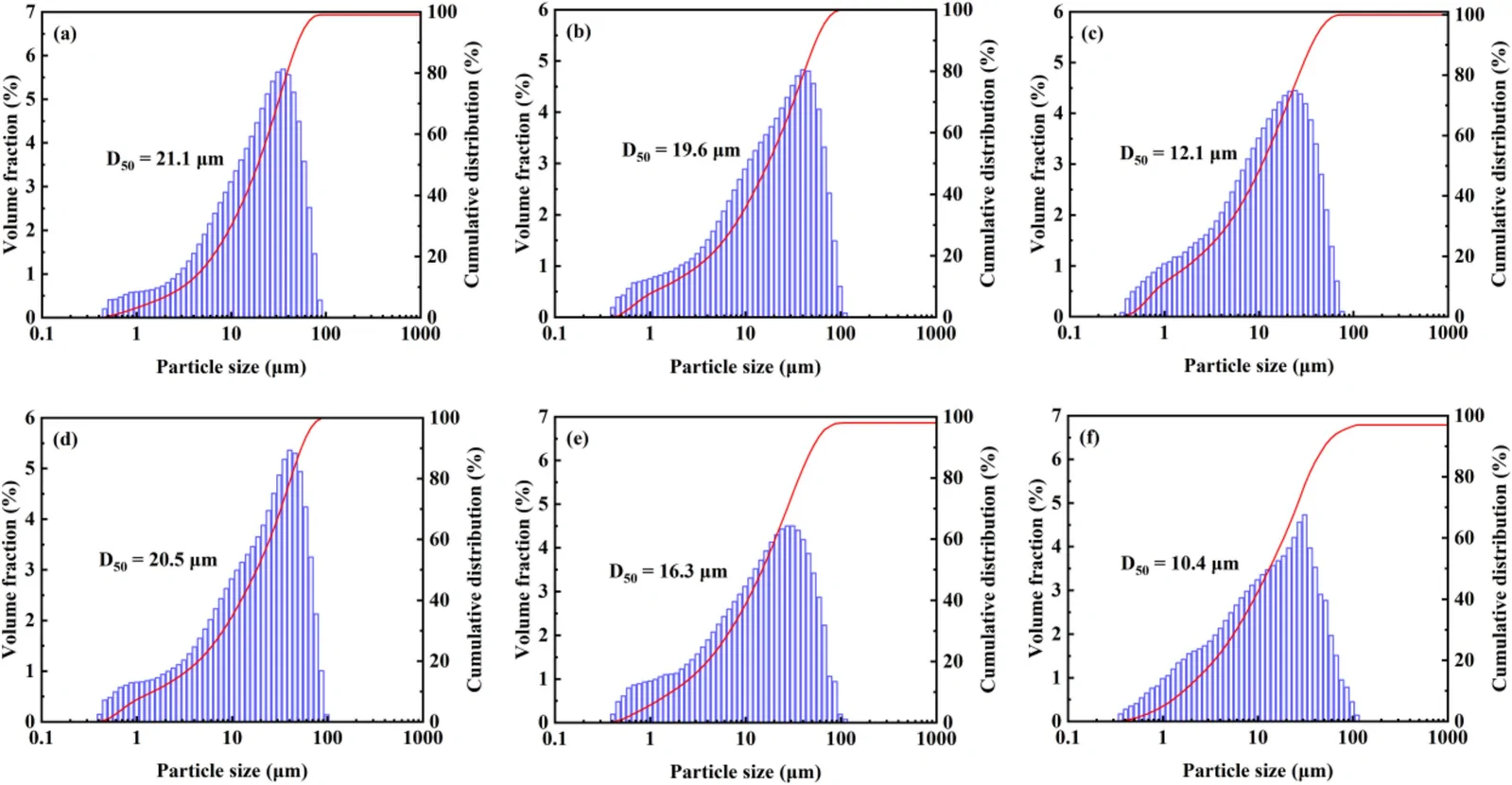
PS distribution of the RVS particles after UT and EUT in cylindrical reactors with different diameters, (a) UT in a cylindrical reactor with a diameter of 8 cm, (b) UT in a cylindrical reactor with a diameter of 6 cm, (c) UT in a cylindrical reactor with a diameter of 4 cm, (d) EUT in a cylindrical reactor with a diameter of 8 cm, (e) EUT in a cylindrical reactor with a diameter of 6 cm, (f) EUT in a cylindrical reactor with a diameter of 4 cm.

Fig. 14 shows that both the UT and the EUT can effectively improve the SSA of the RVS particles with the PS smaller than 74 μm. As shown in Fig. 14(a)–(c), the SSA value of the ultrasound treated RVS increased from 2.31 m^2^ g^−1^ to 4.70 m^2^ g^−1^ with reducing the cylindrical reactor diameter from 8 cm to 4 cm. The fragmentation extent of the RVS minerals was enhanced by inducing electric field into ultrasound field. With reducing the cylindrical reactor diameter from 8 cm to 4 cm, the SSA value of the electric field intensified ultrasound treated RVS increased from 5.84 m^2^ g^−1^ to 9.31 m^2^ g^−1^ (Fig. 14(d)–(f)). Consequently, the reactive surface area of the vanadium-bearing spinel in the RVS can be effectively enlarged by EUT, which is expected to improve the vanadium leaching rate.

**Fig. 14:**
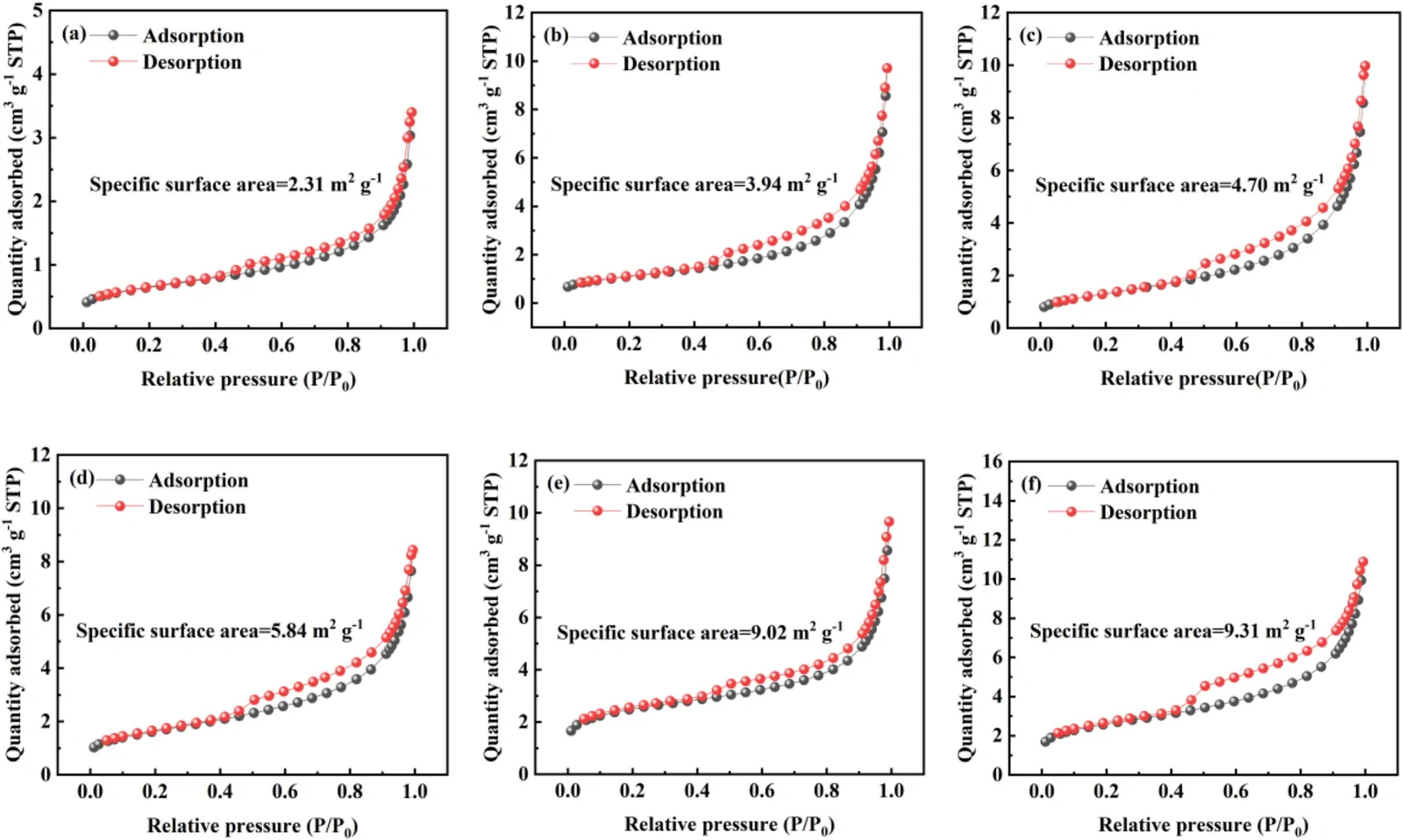
SSA values of the RVS particles after UT and EUT in cylindrical reactors with different diameters, (a) UT in a cylindrical reactor with a diameter of 8 cm, (b) UT in a cylindrical reactor with a diameter of 6 cm, (c) UT in a cylindrical reactor with a diameter of 4 cm, (d) EUT in a cylindrical reactor with a diameter of 8 cm, (e) EUT in a cylindrical reactor with a diameter of 6 cm, (f) EUT in a cylindrical reactor with a diameter of 4 cm.

## 4 Conclusion

The fragmentation behavior of the RVS minerals through EUT in a neutral medium was investigated. The major mineralogical compositions of the RVS were spinel and fayalite, accounting for 90.48 wt% of the total mass. The spinel and fayalite are mechanically connected at the grain boundaries. The silicate-wrapped structure of the spinel can be destroyed through EUT, which enhanced its reactive surface area. Under the action of electric field intensified ultrasound cavitation, the broken extent of the silicates was greater than that of the spinel.

Both the ultrasound and electric field intensified ultrasound impact force increased with decreasing ultrasound WD. Under the action of ultrasound alone and electric field intensified ultrasound, the evolution of ultrasonic impact force on the x-axis and y-axis with ultrasound WD conformed to the natural exponential functional relationship. Inducing electric field into ultrasound field improved the ultrasonic impact force, however, the impact force caused by electric field intensified ultrasound cavitation decayed more rapidly with increasing ultrasound WD. The EUT had little effect on the fragmentation of the RVS particles with PS larger than 74 μm. While, it had significant effect on the reduction in PS and the enhancement of SSA of the RVS particles with PS smaller than 74 μm. The PS of the RVS with PS smaller than 74 μm decreased by 61.2%, while that of the SSA increased by 18.3 times through EUT, which is beneficial to shortening the leaching time and improving the leaching efficiency of vanadium from the RVS.

In practice, the efficiency of ultrasound-assisted leaching can be improved through inducing electric field into ultrasound field. The RVS with PS smaller than 74 μm is recommended to be selected to ensure the sufficient fragmentation of the RVS minerals and the high vanadium leaching efficiency using EUT. The equipment used for extracting vanadium from the RVS via EUT should ensure a sufficiently small reaction space to guarantee the fragmentation effect on the RVS minerals.

## Data availability

Date will be made available on request.

## CRediT authorship contribution statement

**Luyang Duan:** Writing – original draft, Investigation. **Qianqian Ren:** Methodology, Investigation, Funding acquisition. **Shuang Cai:** Resources, Investigation. **Xuelin Ma:** Investigation. **Junguo Li:** Project administration, Methodology, Funding acquisition. **Yanan Zeng:** Resources, Investigation. **Yajun Wang:** Resources, Investigation. **Bao Liu:** Writing – review & editing, Resources, Methodology, Investigation, Funding acquisition.

## Declaration of competing interest

The authors declare that they have no known competing financial interests or personal relationships that could have appeared to influence the work reported in this paper.
